# Graphene Functionalized with Arginine Decreases the Development of Glioblastoma Multiforme Tumor in a Gene-Dependent Manner

**DOI:** 10.3390/ijms161025214

**Published:** 2015-10-23

**Authors:** Ewa Sawosz, Sławomir Jaworski, Marta Kutwin, Krishna Prasad Vadalasetty, Marta Grodzik, Mateusz Wierzbicki, Natalia Kurantowicz, Barbara Strojny, Anna Hotowy, Ludwika Lipińska, Joanna Jagiełło, André Chwalibog

**Affiliations:** 1 Department of Animal Nutrition and Biotechnology, Warsaw University of Life Sciences, Warsaw 02-787, Poland; E-Mails: ewa_sawosz@sggw.pl (E.S.); jaworski.slawek@gmail.com (S.J.); marta_prasek@sggw.pl (M.K.); mgrodka@gmail.com (M.G.); mateusz.wierzbicki1@gmail.com (M.W.); natalia.kurantowicz@gmail.com (N.K.); barb.strojny@gmail.com (B.S.); anna_hotowy@sggw.pl (A.H.); 2 Department of Veterinary Clinical and Animal Sciences, University of Copenhagen, Frederiksberg 1870, Denmark; E-Mail: krish@sund.ku.dk; 3 Institute of Electronic Materials Technology, Warsaw 02-787, Poland; E-Mails: ludwika.lipinska2008@gmail.com (L.L.); joanna.jagiello@itme.edu.pl (J.J.)

**Keywords:** graphene, reduced graphene oxide, amino acids, glioblastoma multiforme, cells, tumor, gene expression

## Abstract

Our previous studies revealed that graphene had anticancer properties in experiments *in vitro* with glioblastoma multiforme (GBM) cells and in tumors cultured *in vivo*. We hypothesized that the addition of arginine or proline to graphene solutions might counteract graphene agglomeration and increase the activity of graphene. Experiments were performed *in vitro* with GBM U87 cells and *in vivo* with GBM tumors cultured on chicken embryo chorioallantoic membranes. The measurements included cell morphology, mortality, viability, tumor morphology, histology, and gene expression. The cells and tumors were treated with reduced graphene oxide (rGO) and rGO functionalized with arginine (rGO + Arg) or proline (rGO + Pro). The results confirmed the anticancer effect of graphene on GBM cells and tumor tissue. After functionalization with amino acids, nanoparticles were distributed more specifically, and the flakes of graphene were less agglomerated. The molecule of rGO + Arg did not increase the expression of *TP53* in comparison to rGO, but did not increase the expression of *MDM2* or the MDM2/TP53 ratio in the tumor, suggesting that arginine may block *MDM2* expression. The expression of *NQO1*, known to be a strong protector of p53 protein in tumor tissue, was greatly increased. The results indicate that the complex of rGO + Arg has potential in GBM therapy.

## 1. Introduction

Primary brain tumors represent about 2% of all malignant tumors in adults; 50%–60% of these are astrocyte gliomas [1]. Although these tumors are relatively uncommon, they unfortunately generate major clinical problems because of their infiltrative growth, aggressive character and progression to malignancy [2]. Glioblastoma multiforme (GBM) is extremely aggressive and the most lethal type of brain tumor. After diagnosis the median patient survival is approximately one year [3,4]. Major challenges in the therapy of GBM are associated with the tumor location within the brain, which greatly complicates surgical removal, and the fact that pharmacological therapy is extremely harmful to healthy tissues [5]. Consequently, research into new methods for GBM therapy that minimize side effects remains indispensable.

Graphene, a single atomic layer of *sp*^2^-bonded carbons [6], and graphene oxide have been recently investigated as nanostructures useful in anticancer treatment. There are two main avenues to this research. The first is focused on the use of graphene as a delivery platform [7,8,9]. The second trend is focused on graphene as a drug-like structure possessing anticancer activity [10,11]. However, the behavior of graphene within an organism is still controversial, regardless of its application.

The most important factor is that the solubility of graphene in water is low [12], which consequently affects the transportation and utilization of graphene within an organism; thus, graphene shows a tendency to agglomerate and form deposits at the site of administration [13]. This characteristic may positively limit the range of graphene penetration into healthy tissue when administered into tumor tissue, but negatively decreases its activity due to a reduced surface area after agglomeration.

Our previous studies revealed that graphene had anticancer properties in *in vitro* experiments with GBM cells and in experiments with GBM tumors cultured *in vivo* [14]. We demonstrated that graphene enters into GMB cells and other cells in GMB tissue, causing severe destruction of cells by triggering apoptosis. However, nanoparticles of graphene deposited within tissue or cells showed a tendency to agglomerate, which probably decreased the graphene-biostructure interface within the tissue and/or cell. We hypothesized that the addition of amino acids to graphene solutions might counteract graphene agglomeration. Moreover, amino acids, which are natural, small molecules involved in specific interactions with other molecules, inside and outside cells, may increase anchoring of graphene in the area of amino acids specific localizations, and prevent agglomeration of graphene. The role of proteins as a cargo for carbon nanotubes, when proteins are enabled as internal loads or for external adsorption of nanotubes, was also suggested [15].

Amino acids show natural affinity for graphene surface, interestingly they bind graphene surface according to the structure of side-chain groups [16]. Proline has a unique structure among the common amino acids, having its side chain cyclized onto the backbone nitrogen atom, which is the main reason why proline is a common binding motif [17]. The unique structure of proline distinguishes it from other amino acids, in terms of chemical stability and inelasticity [18]. Proline is a hydrophobic amino acid capable of binding to aromatic residues [19], which may mediate its affinity for graphene, however, the affinity of proline for graphene is small, compared to other amino acids [16]. The binding of proline to graphene may influence the spread of graphene particles in the tissue and increase resistance to agglomeration; consequently, graphene + proline molecules may occupy a larger area in the tumor tissue. Proline participates in the induction and progression of cellular stress [20,21] and in molecular recognition, particularly in intracellular signaling [19], and also participates in signaling mechanisms, particularly those occurring via protein-protein recognition without a translational pathway [22]. The regulatory effect of proline metabolism is connected to stress dependent on p53 regulation, because the first enzyme in the proline degradation pathway (proline oxidase/proline dehydrogenase) is encoded by p53-induced gene 6 (*PIG6*) and induces metabolic responses under stress conditions. One of the most promising anticancer strategies involves exploring the possibilities of p53 protein activation by blocking its binding to MDM2 [23]. Other key studies [24] concerning polymorphisms of p53 demonstrated that this protein also occurs in a form containing arginine instead of proline in the N-terminal domain, which increases its proapoptotic activity by 15-fold. The results of other studies have suggested that the methylation of arginine may play a role in regulating different biochemical properties of p53 that have downstream consequences on the functional result of the p53 response [25]. Moreover, according to Jeong *et al.* [26] the greatest difference between p53-arginine and p53-proline was demonstrated for the *PERP* gene engaged in cell-cell adhesion and apoptosis. The most common genes that are transcribed more efficiently by the p53-arginine protein than the p53-proline protein are related to apoptotic function (*DR4*, *NOXA*, *PUMA*, and *PIG3*). Other studies have also confirmed the participation of arginine in protecting the redox state in cells [27]. Furthermore, arginine as a source of nitric oxide can modify the formation of reactive forms of oxygen in mitochondria and lead to apoptosis. Interestingly, arginine showed the highest affinity for graphene, in comparison to all amino acids [16].

Therefore, proline or arginine may not only protect graphene against agglomeration but also support its anticancer properties. Proline and arginine are perceived as anti-cancer molecules [21,28,29,30]. In the present study, however, it was investigated as to whether amino acids might play a role as a cargo for graphene, supporting its navigation and deposition in tumor tissue, and hence modulate the toxicity of graphene, and also act as potential anti-cancer agents. We hypothesized that counteracting graphene agglomeration within tissue/cells may increase the range of graphene activity. Therefore, we investigated the other trend in anticancer graphene use, where graphene as an anticancer drug is delivered and distributed by organic compounds (amino acids), which also support the anticancer activity of graphene and create a kind of mutual cooperation.

## 2. Results

### 2.1. Characterization of GO (Graphene Oxide) and rGO (Reduced Graphene Oxide)

**Figure 1:**
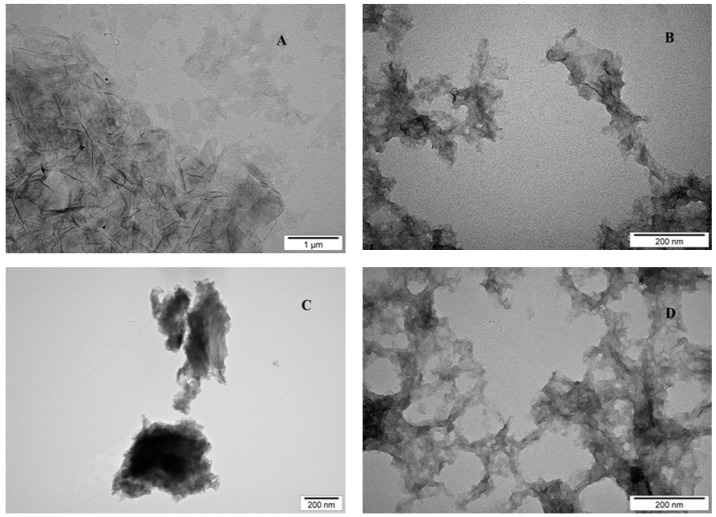
TEM image of graphene oxide (**A**); reduced graphene oxide (**B**); and reduced graphene oxide functionalised with arginine (**C**) and proline (**D**). The ζ potentials were for rGO: 19.5, rGO + Arg: 32.5, rGO + Pro: 39.8, Arg: 9.67 and Pro: 23.8 mV.

Figure 1 shows representative TEM (transmission electron microscope) images of GO (A), rGO (B), rGO + Arg (C), rGO + Pro (D). GO after reduction, a change in morphology was seen, with fewer layers, and irregular and wrinkled flakes, ranging from 100 nm to 1.5 μm in diameter. After functionalization of GO with arginine and proline, graphene and amino acids changed their appearance. Graphene was attached to the amino acids, and there were no visible free flakes of graphene, the structures were irregular and branch like.

**Figure 2:**
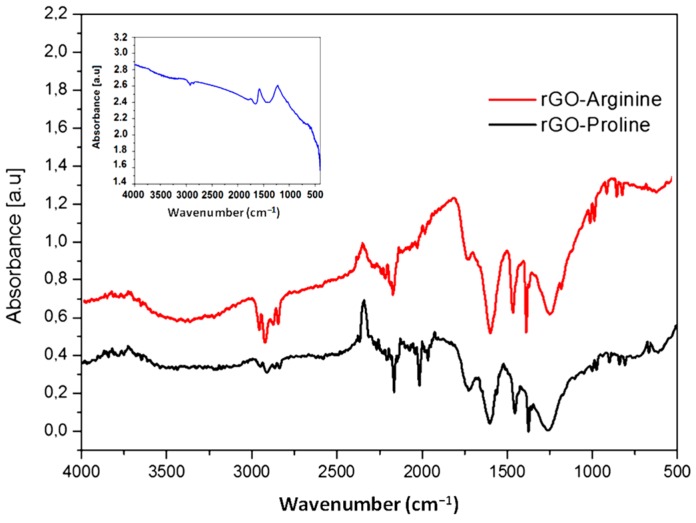
Fourier transform infrared spectroscopy spectra of reduced graphene oxide (top left) and reduced graphene oxide with arginine and with proline.

The Fourier transform infrared spectroscopy (FTIR) spectrum of rGO, rGO + Arg and rGO + Pro samples indicate the presence of amino acids grafted by rGO (Figure 2). Both spectra of graphene functionalized with amino acids are similar and differ from rGO spectra. In the rGO characteristic spectrum, three peaks at 1769, 1602 and 1289 cm^−1^ were observed, corresponding to C=O, C=C and C–O bonds respectively [31]. The spectrum of rGO + Arg and rGO + Pro samples showed presence of groups originated from amino acids functionalization, observed also by the other authors in the spectra of arginine [32], graphene functionalized with poly-l-lysine [33] and graphene functionalized with amine [34]. In the range from 3500 to 3140 cm^−1^, stretching bands derived from O–H group and N–H in free NH_3_^+^ group was observed. At around 1570 cm^−1^ there is out-of-plane bending of NH_2_ group and at around 1236 cm^−1^ overlap C–O and also C–N stretching is seen. Bending vibrations of N–H groups are in the range of 890–810 cm^−1^. At 454 cm^−1^ in the sample with arginine and at 463 cm^−1^ in the sample with proline there are bands due to rocking motion of N–H groups. The C=O stretching vibration, corresponding to carboxylic groups, appears at 1725 cm^−1^.

### 2.2. Experiments with Fibroblast and Glioblastoma Cells

#### Mortality, Viability and Morphology of Cells

**Figure 3:**
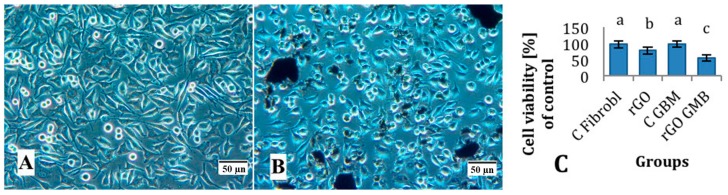
Morphology of L929 fibroblast cells in the control group (**A**); after treatment with reduced graphene oxide (**B**) and the viability of cells (**C**). Notes: Reduced graphene oxide flakes formed agglomerates and adhered to the cells. Bars with different superscripts indicate significant differences between groups (*p* < 0.05); error bars are standard deviations. C Fibrobl: control fibroblasts (untreated); rGO Fibrobl: fibroblasts treated with reduced graphene oxide; C GMB: control glioblastoma (untreated); and rGO GMB: glioblastoma treated with reduced graphene oxide.

In the preliminary experiments with fibroblast L929 the morphology of cells after treatment with rGO differed from the control group. Fewer cells were seen and with graphene agglomerates attached to the cell body (Figure 3B). rGO significantly (*p* < 0.05) decreased viability of both cell lines comparing with the control groups. However, the reduction of GBM cells was significantly higher than fibroblast cells (Figure 3C).

**Figure 4:**
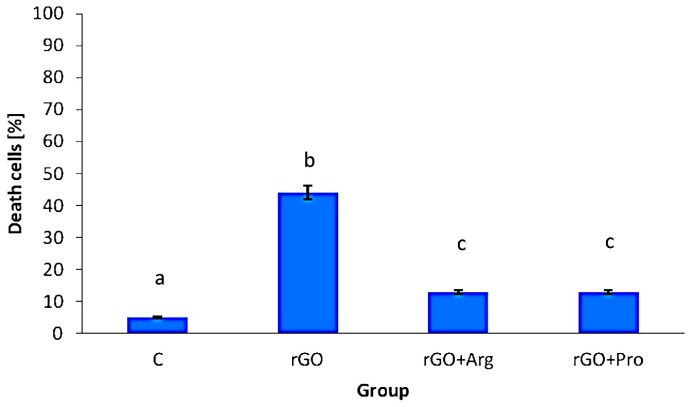
Effect of reduced graphene oxide, reduced graphene oxide with arginine, and reduced graphene oxide with proline on the mortality of glioblastoma U87 cells. Notes: Bars with different superscripts indicate significant differences between groups (*p* < 0.05); error bars are standard deviations. C: control group (untreated cells); rGO: reduced graphene oxide group; rGO + Arg: reduced graphene oxide with arginine group; and rGO + Pro: reduced graphene oxide with proline group.

The measurements of mortality demonstrated that rGO flakes significantly (*p* <0.05) increased the number of dead cells compared with the control group. The complexes of rGO + Arg and rGO + Pro also significantly increased cell mortality but to lesser extent than rGO (Figure 4).

**Figure 5:**
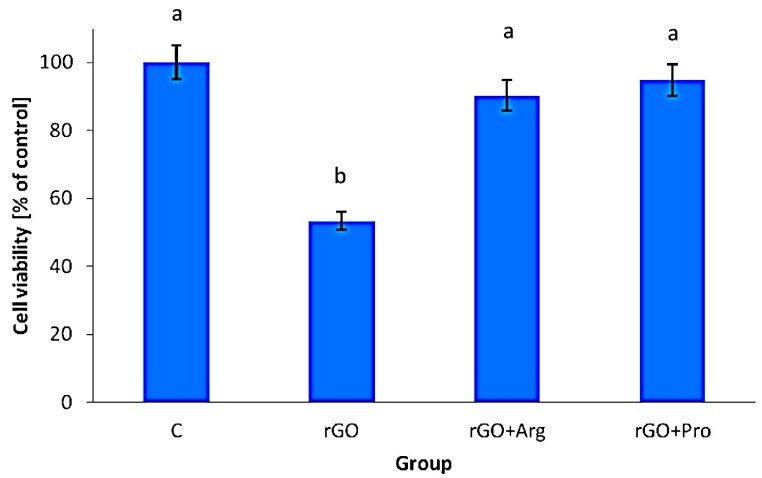
Effect of reduced graphene oxide, reduced graphene oxide with arginine, and reduced graphene oxide with proline on the viability of U87 glioblastoma cells. Notes: Bars with different superscripts indicate significant differences between groups (*p* < 0.05); error bars are standard deviations. C: control group (untreated cells); rGO: reduced graphene oxide; rGO + Arg: reduced graphene oxide with arginine; rGO + Pro: reduced graphene oxide with proline.

After treatment with rGO, the viability of U-87 cells was reduced, but functionalization of graphene with Arg and Pro overcame this negative effect of graphene, and U-87 cell viability was not affected by rGO + Arg and rGO + Pro (Figure 5).

**Figure 6:**
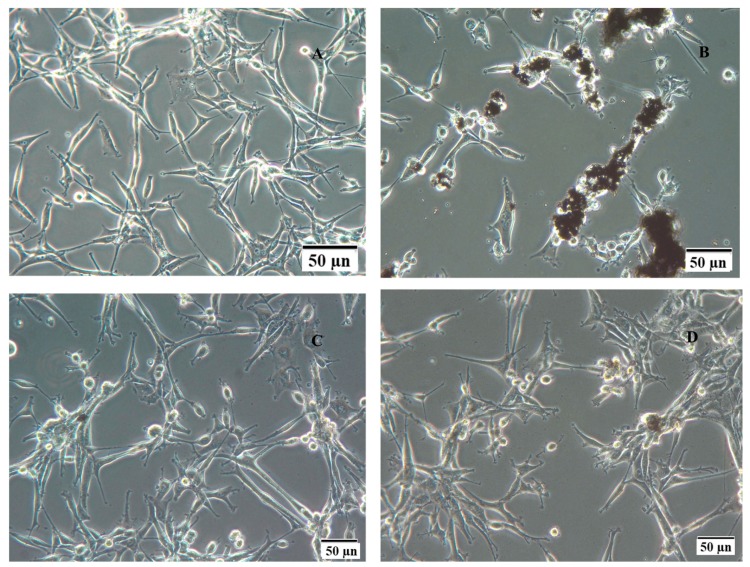
Morphology of U-87 glioblastoma cells in the control group (**A**) and after treatment with reduced graphene oxide (**B**), reduced graphene oxide with arginine (**C**), and reduced graphene oxide with proline (**D**). Notes: Reduced graphene oxide flakes formed agglomerates (**B**), all graphene forms adhered to the cells.

The morphology of GBM cells after treatment with rGO differed from the control. Fewer cells were seen with reduced protrusion and with graphene agglomerates attached to the cell body (Figure 6). rGO + Arg and rGO + Pro did not visibly change the morphology of cells; however, black spots of rGO agglomerates were not seen, and only small shadows were observed on the body of cells. In the group of cells treated with rGO + Arg, graphene was nearly invisible.

### 2.3. Experiments with Tumor Tissue

#### 2.3.1. The Volume, Weight and Morphology of GBM Tumors

GBM U-87 cells were cultured on the chorioallantoic membranes of chicken embryos. The tumor tissue was resected, and the volume, weight and morphology of the tumors were compared (Table 1).

**Table 1:** Characteristics of glioblastoma multiforme U87 tumors cultured on chicken embryo chorioallantoic membrane after treatment with reduced graphene oxide, reduced graphene oxide with arginine and reduced graphene oxide with proline or untreated as a control.

Parameter	Group				ANOVA	
	Control	rGO	rGO + Arg	rGO + Pro	SE	*p*-Value
Weight [mg]	0.0981 ^a^	0.0637 ^b^	0.0666 ^b^	0.0598 ^b^	0.01551	0.0317
Volume [mm^3^]	114.2 ^a^	45.32 ^b^	74.51 ^b^	64.12 ^b^	18.481	0.0049

^a,b^ Values with different superscripts denote a statistically significant difference (*p* < 0.05) between the treatments. Abbreviations: control group (untreated); rGO: reduced graphene oxide group; rGO + Arg: reduced graphene oxide with arginine group; rGO + Pro: reduced graphene oxide with proline group; ANOVA: analysis of variance; and SE: pooled standard error.

**Figure 7:**
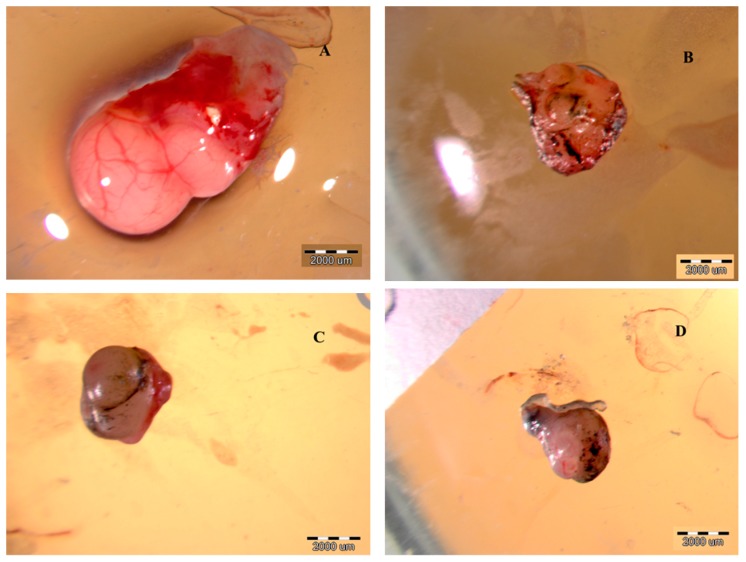
Morphology of U-87 glioblastoma tumors cultured on chicken embryo chorioallantoic membranes in the control group (**A**) and after treatment with reduced graphene oxide (**B**); reduced graphene oxide with arginine (**C**); and reduced graphene oxide with proline (**D**).

rGO and rGO functionalized with arginine and proline significantly reduced the weight of the tumors. The volume of the tumors after treatment with rGO, rGO + Arg, and rGO + Pro was reduced in comparison to the control; however, the greatest reduction was observed with rGO. The morphology of the tumors after treatment with rGO differed from the control (Figure 7).

The solid part of the tumor was less rounded, wrinkled and creased with fewer visible blood vessels. Dark grey or black spots of graphene agglomerates were seen. After treatment with rGO + Arg, solid parts of tumors were smaller and rounded without wrinkles; however, comparing to the other groups, the network of blood vessels reduced to the greatest extent. rGO + Pro also decreased the solid volume of the tumor, but did not change the surface morphology. Graphene particles were easily visible.

#### 2.3.2. Histology of Tumors

**Figure 8:**
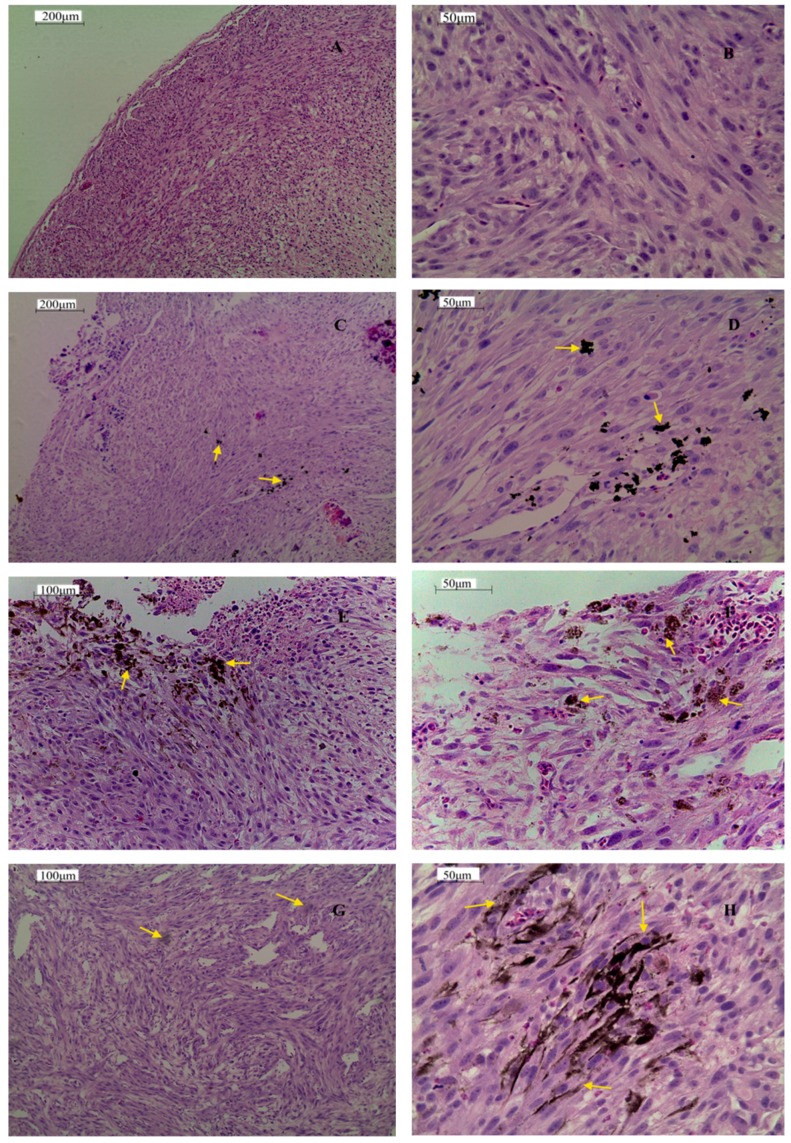
Histology of U-87 glioblastoma tumors cultured on chicken embryo chorioallantoic membranes in the control group (**A**,**B**) and after treatment with reduced graphene oxide (**C**,**D**); reduced graphene oxide with arginine (**E**,**F**); and reduced graphene oxide with proline (**G**,**H**). Arrows point to nanoparticles. Note: Reduced graphene oxide flakes formed agglomerates.

Pictures of the control GBM tumor presented a typical microstructure (Figure 8). Two basic morphologic features were seen: necrosis and endothelial proliferation. Centrally located necrosis and palisading cells around necrotic foci were observed. Formations of blood vessels were also noted, mainly in the outer layer of the tumor. The presence of pink fibrillary cytoplasm in the cells was also seen. Images of the GBM tumors treated with rGO indicated the presence of graphene agglomerates within the tissue. rGO was randomly distributed in the central area of the tumor as well as in the core. The tissue, however, was full of white gaps and ruptures. GBM tumors, after injection with rGO + Arg and rGO + Pro, appeared different. In the rGO + Arg group, graphene was placed on the outer layer of the tumor, slightly aligned and often located close to small blood vessels and in microglia cells. In the tumors treated with rGO + Pro, the agglomeration of graphene particles was greatly reduced, and the particles were aligned in the tissue and found between cells and around cells rather than inside the cells.

#### 2.3.3. Gene Expression in Tumors

Expression of the tumor protein p53 (*TP53*), at the mRNA level, was increased in tumors after treatment with rGO; however, this effect was diminished by amino acid attachment, but the level of *TP53* after rGO + Arg treatment was higher in comparison to the control (Table 2).

The expression of mouse double minute 2 (*MDM2*) was significantly higher in tumors treated with rGO in comparison to the control. Graphene functionalized with arginine and proline diminished this property; however, the expression of *MDM2* was slightly but non-significantly higher than in the control group. The *MDM2/TP53* ratio was lowest in the rGO + Arg group. The expression of cytochrome c oxidase 6 (*COX6*) increased after treatment with all experimental treatments, with the highest expression found in rGO and rGO + Arg treated tumors. NAD(P)H: quinone acceptor oxidoreductase 1 (*NQO1*) expression was increased after injection of rGO, even more after treatment with rGO + Pro and much more after rGO + Arg treatment. The expression of caspase-3 (*CASP3*) was increased in all experimental groups; however, this was significant only in the groups treated with rGO and rGO + Arg. Expression of fibroblast growth factor-2 (*FGF2*) was lower in all experimental groups than in the control. The mRNA level of vascular endothelial growth factor A (*VEGF*) was slightly (non-significantly) lower in tumors injected with rGO + Arg.

**Table 2:** Gene expression at the mRNA level in glioblastoma multiforme U87 tumors, cultured on chicken embryo chorioallantoic membrane, after treatment with reduced graphene oxide, reduced graphene oxide with arginine and reduced graphene oxide with proline or untreated as a control.

Genes	Group				ANOVA	
	Control	rGO	rGO + Arg	rGO + Pro	SE	*p*-Value
*TP53*	0.345 ^a^	1.310 ^b^	0.965 ^c^	0.604 ^a^	0.0999	0.0001
*MDM2*	0.845 ^a^	3.053 ^b^	1.919 ^a^	1.564 ^a^	0.3544	0.0065
*MDM2/TP53*	2.45 ^a^	2.33 ^a^	1.99 ^b^	2.59 ^a^	0.288	0.0032
*COX6*	0.323 ^a^	0.950 ^c^	1.082 ^c^	0.714 ^b^	0.0777	0.0001
*NQO1*	0.380 ^a^	3.781 ^b^	10.845 ^c^	5.012 ^b^	0.2208	0.0000
*CASP3*	0.428 ^a^	0.920 ^b^	1.052 ^b^	0.780 ^a,b^	0.1724	0.0115
*FGF2*	1.059 ^a^	0.671 ^b^	0.432 ^b^	0.741 ^b^	0.0956	0.0045
*VEGF*	0.528	0.438	0.287	0.526	0.1723	0.1156

^a,b,c^ Values with different letters denote a statistically significant difference between the groups. Abbreviations: control group (untreated); rGO: reduced graphene oxide group; rGO + Arg: reduced graphene oxide with arginine group; rGO + Pro: reduced graphene oxide with proline group; ANOVA: analysis of variance; and SE: pooled standard error.

## 3. Discussion

In the present studies we measured both mortality and viability of cells. The number of dead cells is a measurement of the integrity of the cell membrane, while viability is related to NAD(P)H production through glycolysis and correlates to the number of metabolically active cells in the culture. The results with experiments with GBM cells confirmed our earlier studies indicating that rGO increases cell mortality [10,14,35]. The toxic effects of rGO in *in vitro* experiments with normal and cancer cells have been demonstrated previously [36,37,38,39]. However, the toxic effects also depend on the type of the cells, as it was observed that fibroblasts (Figure 3), being less susceptible to the rGO treatment than GBM. This might be explained by the high amount of graphene aggregates in fibroblast medium and lower affinity of the rGO flakes to fibroblast cells, influencing adhesion to the cell membrane and rGO intake by cells. Although, the lower toxicity of rGO to fibroblasts than to GBM might be considered, the method of potent use of graphene as an anticancer structure presupposes direct intratumor injections.

The toxic effect of graphene can be explained by the interaction of hydrophobic chemically active groups, available on the surface, with the membranes of living cells, as well as other intracellular structures [36,37,38,39]. However, the toxicity of graphene may be reduced after the adsorption of amino acids onto graphene sheets [40]. The treatment with rGO after functionalization with amino acids led to significantly reduced number of dead cells compared to rGO. However, still the toxicity of rGO + Pro and rGO + Arg was higher than in the control group. The harmful effects of graphene might be mediated by hydrophobic groups exposed on the rGO surface, which became unavailable after Pro and Arg functionalization. In other experiments using graphene oxide (GO), the toxicity was greatly mitigated following extremely high protein adsorption on GO, where proteins, as a crown placed on graphene, decreased the number of active groups available on the surface [41].

Visualization of the morphology of the graphene-GBM cell interaction, as in our previous experiments, pointed to the affinity, adherence and entry of rGO into cells [10]. The phenomenon of graphene transport into cells has also been documented in other studies [13,14]. Functionalization of rGO by amino acids (Pro and Arg), however, dramatically changed rGO behavior. Graphene flakes, after administration to cultured cells, adhered to the body of cells, agglomerated, and were seen as black spots. Visualization of rGO + Arg and rGO + Pro did not show this kind of structure, and agglomerates of graphene were not seen clearly as black spots. This might suggest that the functionalization of rGO with Pro and Arg prevented rGO agglomeration. Shan *et al.* [33] demonstrated increased solubility of graphene after functionalization of Poly-l-lysine, what also might occur in the present experiment, especially that Zeta potential of rGO after functionalization with amino acids increased about two-fold.

The results on cell viability may partially explain the above observations. rGO treatment induced significant cell toxicity, as well as decreased viability, but rGO + Pro and rGO + Arg only affected the mortality of GBM cells. We suspect that diminished agglomeration due to arginine and proline functionalization inhibited the entry of graphene into cells, but did not affect the intracellular mechanism, *i.e.*, NAD(P)H production. The number of dead cells was reduced, but the toxicity of rGO + Arg and rGO + Pro was higher than in the control group, mediated by the destruction of cell membranes due to graphene flakes sticking to the membranes. It is likely that amino acids increased the adhesion of flakes to the membranes, and thereby increased their toxicity.

In the experiment with GBM tumors cultured on the chicken embryo chorioallantoic membranes, rGO decreased the weight and volume of the tumors. The decreased tumor volume was accompanied by reduced tension of the tumor tissue, manifested by wrinkling of the surface. In our previous study, apoptosis of cancer cells was observed after graphene treatment [14], resulting in a reduction in tumor weight and volume. However, a disruption in water homeostasis might also occur, especially since graphene is permeable to water but not permeable to ions [42]; thus, graphene could act as a kind of filter, as was also suggested by Jaworski *et al.* [14] The volume of the tumors in rGO + Arg and rGO + Pro groups was smaller than in the control group but greater than in the rGO group, which was also confirmed by the more wrinkled surface of the tumor treated with rGO. Consequently, this may suggest that the proliferation of cells was decreased by all graphene treatments (rGO, rGO + Arg and rGO + Pro), but water circulation was changed only by rGO.

The decreased rate of cell proliferation in the tumors after all treatments was confirmed by *FGF2* expression on the mRNA level. FGF2 is a marker of cell proliferation during tumor development; moreover, the dramatic effects of FGF2 in cancer result from FGF2-induced shifts in gene expression [43]. FGF2 is necessary for maintaining VEGFR2 receptor, hence, the inhibition of FGF2 downregulates VEGF-dependent biological processes, mainly angiogenesis [44]. In the present study, we observed a tendency for decreased *VEGF* expression only after rGO + Arg treatment. This result is in line with the examination of the histology images from the rGO + Arg group, where graphene was found mainly in the outer, most metabolically active layer of the tumor, slightly aligned and often located near small blood vessels. This may suggest that arginine was preferentially localized in these areas. Tumor downregulation of the argininosuccinate synthetase results in a dependence on extracellular arginine, due to an inability to synthesize arginine for growth [45]. Thus, the requirement of cancer cells for arginine may have increased the movement of rGO + Arg molecules preferentially to the areas of most aggressive tumor growth. Consequently, graphene was localized in the outer layer of the tumor, in the area of the highly active process of angiogenesis, in contrast to rGO and rGO + Pro.

Our previous studies did not document any anti-angiogenic effects of graphene [46], suggesting that the antiangiogenic effect observed in this study was induced by arginine, which was protected from breakdown by the association with graphene. However, the involvement of arginine in angiogenesis is controversial [47,48,49].

In the present experiment, the mRNA level of *NQO1* increased dramatically after rGO + Arg treatment and to a lesser extent by rGO and rGO + Pro treatments. NQO1 promotes the two-electron reduction of quinones, nitroaromatics and quinoneimines and consequently depresses the quinone level, which minimizes the generation of reactive oxygen species [50]. Graphene-like materials, however, can protect molecular targets from oxidation by free radicals, and are highly effective scavengers of hydroxyl radical [51]. Furthermore, NQO1 plays a broad role in cytoprotection because it binds to and consequently stabilizes the tumor suppressor p53 protein from proteasomal degradation [52]. In the present experiments, however, the expression of *TP53* at the mRNA level also increased after rGO + Arg and to a higher degree after rGO application. Furthermore, rGO upregulated the expression of *TP53*, indicating a dual role of rGO by stimulation of the *TP53* gene and by stimulation of the protector of p53, *NQO1*. As a key tumor suppressor, p53 inhibits tumorigenesis by inducing cell cycle arrest, senescence, and apoptosis [53]. Apoptosis of GMB cells as a result of rGO treatment was observed in our earlier experiments [10,14]. The present experiments confirm these results, demonstrating the upregulation of *CASP3*, which plays a key role in the execution phase of apoptosis.

During stress, the expression of p53 increases in glioma [54], but its actual activity is determined by the level of MDM2, which binds to the N-terminal domain of p53 and blocks its proapoptotic activity [55]. Some authors believe that blocking the binding of p53 with MDM2 by selective blocking of the MDM2 binding domain without deregulation and deactivation of p53 protein could benefit anticancer therapy [56,57]. In all experimental groups, increased expression of *MDM2* at the mRNA level was observed. However, the MDM2/TP53 index was significantly lower only in rGO + Arg treated tumors. This raises the issue that only the interaction of graphene and arginine may modify molecular responses in the most promising direction.

TP53 has several polymorphisms, including a proline to arginine variant at amino acid 72 (P72 to R72). The P72 variant induces cell cycle arrest, while the R72 variant has the ability to preferentially induce apoptosis [53]. Thus, we can hypothesize that an additional reservoir of arginine, provided and protected against enzymatic digestion by the rGO platform [58], may support the synthesis of DNA for properly encoded TP53 with the R72 polymorphism (arginine variant). This is in line with the results concerning CASP3-mediated activation of apoptosis.

Considering the results of rGO + Pro administration in comparison to rGO + Arg, the MDM2/TP53 index and reduced expression of *NQO1* indicate the insufficient effect of this complex. The data may also confirm that, in the case of TP53 with the R72 polymorphism, supplementation with proline has no impact on the mRNA expression of *TP53*.

Interestingly, *COX6* mRNA expression was significantly induced by rGO and rGO + Arg and to a lesser degree by rGO + Pro. The intrinsic pathway of apoptosis occurs by releasing COX6 into the cytosol, which then causes the assembly of a multiprotein caspase-activating complex [59]. p53 activates this mechanism in a transcriptionally-dependent or -independent manner [60]. In our experiments, the activation of *TP53* and *COX6* transcription was similar, *i.e.*, higher in the rGO and rGO + Arg treatments and lower in the control and rGO + Pro groups. Considering these results, as well as decreased *CASP3* expression mediated by rGO + Pro, may indicate that proline attached to graphene reduces the proapoptotic activity of rGO.

## 4. Experimental Section

### 4.1. Preparation of Graphene Complexes

Graphene oxide (GO) was prepared from natural graphite flakes (purchased from Asbury Carbons, Asbury, NJ, USA) by a modified Hummers method [14]. To prepare the reduced graphene oxide (rGO), a water suspension of 50 mg of GO was acidified to pH 1 and heated to 90 °C. In the next step, 12 mL of reducing mixture (0.01 g of ammonium iodide, 9 g of hydrated sodium hypophosphite, and 1.21 g of sodium sulfite dissolved in 100 mL of deionized water) was added. A black material (rGO) precipitated immediately. The product was filtered, washed with deionized water, and dried. The rGO powders were used to make aqueous suspensions for further analysis and applied in experiments. The aqueous suspensions were done by adding the powder to ultrapure water and sonicating the solution at 550 W/m^2^ for 1 h. To prepare graphene + l-arginine (rGO + Arg) or graphene + l-proline (rGO + Pro) complexes, the amino acid (mass ratio of 1:1) was added to the GO aqueous suspension and mixed on a magnetic stirrer to dissolve the amino acid. Then, a mixture of reducers, *i.e.*, sodium hypophosphite (0.5 g) and hydroiodic acid (5 mL at a concentration of 57%) was added. The process of reduction was conducted for approximately 3 h at a temperature of 80 °C. At the end of the procedure, gentle sonication of the sample was performed. The procedure was the same with each of the amino acids (arginine and proline). l-arginine and l-proline were obtained from Sigma-Aldrich (St. Louis, MO, USA). In the preliminary tests we have established the quantity of amino acids, linked with graphene without leaving residues of graphene, as 1:1 proportion of amino acid to graphene. Ninhydrine (2,2-Dihydroxyindane-1,3-dione) was used to detect amino acids in the washed out fluid.

The size and shape of the graphene sheets were examined by a JEM-1220 (JEOL, Tokyo, Japan) transmission electron microscope (TEM) at 80 KeV, with a Morada 11-megapixel camera (Olympus Soft Imaging Solutions, Münster, Germany). The samples for TEM were prepared by placing droplets of hydrocolloids on to Formvar-coated copper grids (Agar Scientific, Stansted, UK). The droplets were dried in dry air, and immediately the grids were inserted into the TEM. The tests were performed in triplicate.

The ζ potential in water was measured by a Zetasizer Nano ZS model ZEN3500 (Malvern Instruments, Malvern, UK). The FTIR spectra were measured by Vertex 80v (Bruker BioSpin Corporation, Billerica, MA, USA) in the range 500 to 4000 cm^−1^.

### 4.2. Cell Culture

The human glioblastoma U87 and fibroblast L929 cell lines were purchased from the American Type Culture Collection (Manassas, VA, USA) and maintained in Dulbecco’s Modified Eagle’s culture medium containing 10% fetal bovine serum (Life Technologies, Houston, TX, USA) and 1% penicillin and streptomycin (Life Technologies) at 37 °C in a humidified atmosphere of 5% CO_2_/95% air in a DH AutoFlow CO_2_ air-jacketed incubator (NuAire, Plymouth, MN, USA).

### 4.3. Cell Morphology

U87 glioma cells were seeded in six-well plates (1 × 10^5^ cells per well) and incubated for 24 h. Cells cultured in the medium without any treatment were used as the control group. Graphene and amino acid complexes, at a concentration of 50 μg/mL of rGO and 50 μg/mL of arginine or proline, were introduced to the cells. 24 h after exposure, an optical microscope was used to evaluate cell morphology.

### 4.4. Cell Mortality

The trypan blue assay (Sigma-Aldrich, St. Louis, MO, USA) was used to evaluate cell mortality. U87 cells were plated in 96-well plates (5 × 10^5^ cells per well) and incubated for 24 h. The medium was removed, and rGO, rGO + Arg, or rGO + Pro samples were introduced to the cells. Then, the cells were detached with 300 μL of a trypsin-ethylenediaminetetraacetic acid (EDTA) solution. The mixture was centrifuged at 1200 rpm for 3 min. In the next step, 700 μL of trypan blue solution was added to each well and dispersed. After 5 min, the cells were counted using a CellCounter (Roche, Penzberg, Germany). Dead cells were stained blue. Cell mortality was expressed as the percentage of the dead cells in proportion to the total cell number.

### 4.5. Cell Viability

Cell viability was determined using a 2,3-bis-(2-methoxy-4-nitro-5-sulphophenyl)-2*H*-tetrazolium-5-carboxyanilide salt (XTT)-based cell proliferation assay kit (Life Technologies, Taastrup, Denmark). U87 were plated in 96-well plates (5 × 10^3^ cells per well) and incubated for 24 h. The medium was then removed, and rGO and rGO + Arg, or rGO + Pro samples were introduced to the cells (at the concentration described above). In the next step, 50 μL of XTT solution was added to each well and incubated for an additional 3 h at 37 °C. The optical density of each well was recorded at 450 nm on an enzyme-linked immunosorbent assay reader (Infinite M200, Tecan, Durham, NC, USA). Cell viability was expressed as the percentage (OD_test_ − OD_blank_)/(OD_control_ − OD_blank_), where OD_test_ is the optical density of cells exposed to GO and rGO, OD_control_ is the optical density of the control sample, and OD_blank_ is the optical density of wells without glioma cells. The same procedure was used with fibroblasts L929 and glioblastoma U87 cells treated with rGO.

### 4.6. Culture of GMB on a Chorioallantoic Membrane

The fertilized eggs (*Gallus gallus*; *n* = 80) were obtained from a commercial hatchery (Dębówka, Poland). After 6 days of incubation (under standard conditions), a silicone ring ×10 containing 3–4 × 10^6^ U87 cells, suspended in 30 μL of culture medium, was placed on the chorioallantoic membrane. The eggs were incubated for the following 7 days, and then randomly divided into four groups (*n* = 20 each): control not injected and rGO, rGO + Arg and rGO + Pro, injected with 200 μL of each solution. The concentration of rGO, rGO + Arg or rGO + Pro was 500 μL of rGO and 500 μL of arginine or proline. The solutions were directly injected into the tumor tissue. After 3 days, the tumors were resected for pending analysis.

### 4.7. Tumor Volume and Histology

A stereomicroscope (SZX10, CellD software version 3.1; Olympus Corporation, Tokyo, Japan) was used to take digital photographs of the tumors. The measurements were performed with cellSens Dimension Desktop version 1.3 (Olympus). The tumor volumes were calculated using the method described by Jaworski *et al.* [14].

After resection, tumors were fixed in 4% buffered formalin (Sigma-Aldrich, St. Louis, MO, USA). Samples were dehydrated and embedded in paraffin. Sections 5 μm in thickness were placed on poly-l-lysine-coated slides (Equimed, Krakow, Poland) and stained with hematoxylin and eosin. The measurements were carried out using an optical microscope DM750; Leica Microsystems GmbH, (Wetzlar, Germany) and LAS EZ version 2.0 software (Wetzlar, Germany). Morphometric estimation and image analysis were made using 20 measurements of each sample at 400× magnification. The mitotic index was evaluated as the number of mitotic figures in ten visual fields.

### 4.8. Gene Expression at the mRNA Level

The tumor tissue samples were homogenized in TRIzol^®^ Reagent (Thermo Fisher Scientific, Waltham, MA, USA), and total RNA was extracted according to the manufacturer’s instructions. The RNA was purified using the SV Total RNA Isolation System (Promega Corporation, Madison, WI, USA) and quantitated using a NanoDrop spectrophotometer (NanoDrop Technologies, Wilmington, DE, USA). Quality was further measured using an Agilent 2100 bioanalyzer (Agilent RNA 6000 Nano kit, Waldbronn, Germany). The RNA integrity number (RIN) higher than 6.5 was considered acceptable to proceed with complementary DNA (cDNA) synthesis. Using 2 µg of total RNA was reverse transcribed for cDNA synthesis, after which real-time PCR was performed with cDNA and the gene specific primers shown in Table 3 (TAG, Copenhagen A/S, Copenhagen, Denmark) mixed with SYBR Green Master mix (Roche Applied Science, Penzberg, Germany) in a Light Cycler^®^ 480 real-time PCR system (Roche Applied Science, Penzberg, Germany). The cycling conditions included with an initial denaturing step at 95 °C for 15 min, followed by 40 cycles with a denaturing step at 94 °C (15 s), an annealing step at 56 °C (30 s), and an elongation step at 72 °C (60 s). The cycling reports and melting curves were evaluated as part of the analysis. Each individual sample reaction was performed in triplicate. For analyses, relative quantification was calculated *versus* expression of the *β actin* (*ACTB*) reference gene.

**Table 3:** Primers used in the study.

Gene	Forward Primer (5′–3′)	Reverse Primer (5′–3′)
*TP53*	CCCAGCCAAAGAAGAAACCA	TTCCAAGGCCTCATTCAGCT
*MDM2*	CAGGACATCTTATGGCCTGCTT	GGGCAGGGCTTATTCCTTTT
*COX6*	TGAATCCGGGGTGCCTTTAG	CAGAGGGACTGGTACACACG
*NQO1*	AGGCTGGTTTGAGCGTGTTC	TTGAATTCGGGCGTCTGCTG
*CASP3*	ACATGGCGTGTCATAAAATACC	CACAAAGCGACTGGATGAAC
*FGF2*	GGCACTGAAATGTGCAACAG	TCCAGGTCCAGTTTTTGGTC
*VEGFA*	TGAGGGCCTAGAATGTGTCC	TCTTTTGACCCTTCCCCTTT
*ACTB*	ACCCAGATCATGTTCGAGACCTT	TCACCGGAGTCCATCACGAT

Abbreviations: *TP53*, tumor protein p53; *MDM2*, mouse double minute 2; *COX6*, cytochrome c oxidase subunit VIb polypeptide 1; *NQO1*, NAD(P)H: quinone acceptor oxidoreductase 1; *CASP3*, caspase-3; *FGF2*, fibroblast growth factor-2; *VEGFA*, vascular endothelial growth factor A; *ACTB*, β-actin.

## 5. Conclusions

This study confirmed the anticancer effect of reduced graphene oxide on glioblastoma multiforme cells, cultured *in vitro* as well as in tumor tissue *in vivo*. Moreover, rGO activated *TP53* gene expression, which is responsible for cellular protection and the stress response, and consequently increased the expression of *COX6* and *CASP3* involved in apoptosis. Interestingly, the *NQO1* gene, responsible for quinone and nitroaromatic reduction and protection of p53 from proteasomal degradation, was activated. This may indicate a new mechanism of the anticancer activity of graphene. However, flakes of rGO after introduction to cells were agglomerated and randomly spread, which decreased its surface area and interfered with the distribution of rGO within the tumor.

After functionalization with amino acids, rGO was distributed more specifically, and flakes of graphene were less agglomerated. Undoubtedly, amino acids directed the distribution of graphene within the tumor, playing the role of tailoring molecules. Moreover, arginine but not proline enhanced the anticancer activity of rGO at the molecular level. The rGO + Arg molecule, however, did not increase the expression of *TP53* in comparison to rGO, but also did not increase the expression of *MDM2* (a key protein in binding and deactivating p53) or the *MDM2/TP53* ratio in the tumor, suggesting that arginine may block *MDM2* expression. Furthermore, rGO + Arg did not diminish *COX6* and *CASP3* mRNA expression, which were increased by rGO treatment, indicating that the pro-apoptotic character of rGO was not reduced by arginine functionalization. The most important result was that rGO + Arg strongly increased the expression of *NQO1* in tumor tissue, which may have decreased the generation of reactive oxygen.
